# Effects of Pulmonary Vein Isolation for Atrial Fibrillation on Skin Sympathetic Nerve Activity in Association with Left Atrial Remodeling

**DOI:** 10.3390/jcdd12040123

**Published:** 2025-03-30

**Authors:** Yoichiro Nakagawa, Takashi Kusayama, Mayumi Morita, Yuta Nagamori, Kazutaka Takeuchi, Shuhei Iwaisako, Toyonobu Tsuda, Takeshi Kato, Soichiro Usui, Kenji Sakata, Kenshi Hayashi, Masayuki Takamura

**Affiliations:** 1 Department of Cardiovascular Medicine, Graduate School of Medical Sciences, Kanazawa University, 13-1 Takaramachi, Kanazawa City 920-8641, Japan; yoichiro.n.99@gmail.com (Y.N.);; 2 Department of Health Sciences, Graduate School of Medical Sciences, Kanazawa University, 13-1 Takaramachi, Kanazawa City 920-8641, Japan

**Keywords:** atrial fibrillation, left atrial remodeling, pulmonary vein isolation, skin sympathetic nerve activity

## Abstract

Pulmonary vein isolation (PVI) is an established treatment for atrial fibrillation (AF). While it is known to affect the autonomic nervous system, the relationship between left atrial (LA) remodeling and PVI-mediated neuromodulation remains unclear. We aimed to assess the neuromodulatory effects of PVI using skin sympathetic nerve activity (SKNA). SKNA was recorded one day before and 2–3 days after PVI in 28 paroxysmal AF (PAF) and 33 persistent AF (PerAF) groups. Baseline low frequency to high frequency (LF/HF) ratio was higher in the PAF group (1.23 [interquartile range {IQR}: 0.79–1.76] vs. 0.74 [IQR: 0.49–1.38], *p* = 0.017). After PVI, the PAF group demonstrated significant reductions in burst amplitude (1.46 [IQR: 1.04–2.84] vs. 1.09 [IQR: 0.78–2.17] μV, *p* = 0.015) and LF/HF ratio (0.91 [IQR: 0.73–1.52] vs. 0.71 [IQR: 0.48–1.21], *p* = 0.012), whereas the PerAF group exhibited no such changes. A weak positive correlation was observed between the percentage change in LF/HF ratio and LA volume index in the PAF group (r = 0.572, *p* = 0.002). PVI significantly decreased SKNA in PAF patients but not in PerAF. LA remodeling may hinder the effectiveness of PVI-mediated neuromodulation.

## 1. Introduction

Atrial fibrillation (AF) is the most prevalent arrhythmia, and it is associated with an increased risk of all-cause mortality and complications [1]. Pulmonary vein isolation (PVI) is an established treatment strategy that modifies the triggers of AF. In contrast, AF patients with advanced left atrial (LA) remodeling have a high recurrence rate after PVI [2]. This fact represents a clinical challenge to overcome in AF treatment.

Abnormalities in the cardiac autonomic nervous system (ANS) are thought to be deeply involved not only in AF triggering but also in its maintenance [3]. Additionally, cardiac ANS dysfunction may be exacerbated by AF [4]. Stellate ganglion nerve activity (SGNA) can be estimated by noninvasively recording the electrocardiogram (ECG) and skin sympathetic nerve activity (SKNA) [5]. This new method is called neuECG. Previous studies revealed that SKNA was associated with the temporal clustering of AF and heart rate (HR) acceleration during AF [6,7]. LA enlargement has been described to be associated with AF progression [8], partly due to indirect effects on the afferent cardiac ANS [9]. While PVI may modulate the cardiac ANS by influencing the ganglionated plexus (GP) on the epicardial surface [10], PVI transiently attenuates sympathetic nerve activity (SNA) in relation to LA volume [11,12]. However, the acute effects of PVI-mediated neuromodulation in patients with varying LA remodeling remain unclear.

We hypothesized that the advanced LA remodeling inhibits the neuromodulatory effects of PVI in the acute phase, leading to poorer outcomes. The present study aimed to investigate the acute neuromodulatory effects of PVI on the cardiac ANS using SKNA in AF patients and its association with LA remodeling.

## 2. Materials and Methods

### 2.1. Participants

The present study enrolled consecutive patients who underwent initial PVI using radiofrequency ablation (RFA) or cryoballoon ablation (CBA) for symptomatic AF at Kanazawa University Hospital from April 2021 to March 2024. AF diagnosis and management were performed following the statement from the 2023 American College of Cardiology/American Heart Association/American College of Clinical Pharmacy/Heart Rhythm Society Guideline [13]. The guideline defined paroxysmal AF (PAF) as AF that is intermittent and terminates within 7 days of onset, and persistent AF (PerAF) as AF that continues beyond 7 days. Exclusion criteria were <18 years of age, decompensated heart failure, prior cardiac surgery, pacemaker implantation, and severe valvular disease.

### 2.2. Pulmonary Vein Isolation

All PVI procedures were performed under sedation with dexmedetomidine hydrochloride and fentanyl citrate, and respiratory support was provided by noninvasive positive-pressure ventilation. PVI lesions in RFA were developed with a high power and short duration (40–50 W, 10–30 s) point-by-point ablation strategy. Catheter manipulation was performed under a three-dimensional electroanatomical mapping system (CARTO^®^ [Biosense Webster, Inc., Irvine, CA, USA] or EnSite^TM^ X [Abbott, Plymouth, MN, USA]). PVI lesions with the CBA were developed using second-generation Arctic Front Advance^®^ (Medtronic, Inc., Minneapolis, MN, USA) with intra-catheter temperatures of approximately −50 °C delivered to each pulmonary vein without a mapping system. PerAF patients underwent electrical cardioversion at the end of the procedure to restore sinus rhythm. Bilateral PVI was completed in all cases, and serious complications, such as cardiac tamponade, were not observed.

### 2.3. Echocardiography

All participants underwent echocardiography to confirm cardiac function before PVI. Examiners who were not informed of the details of the PVI procedure performed the examination. Simpson’s modified method in the apical two- and four-chamber view at end-systole, indexed for body surface area (LA volume index [LAVI]), was used to measure the LA volume.

### 2.4. Study Protocol

**Figure 1:**
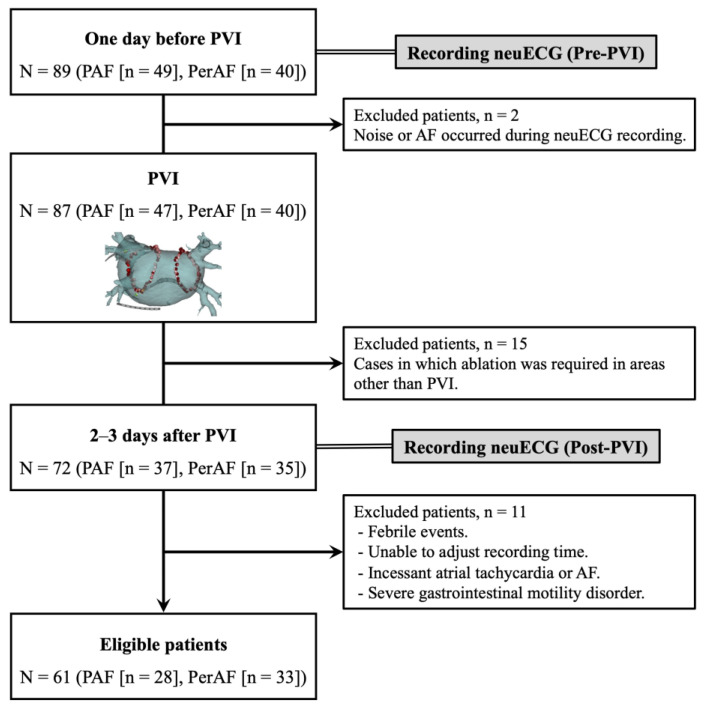
Flowchart of the present study.

In a total of 89 AF patients (PAF [n = 49] and PerAF [n = 40]), neuECG recordings were performed in the resting state for 30 min each before and after PVI (Figure 1). The aim of the present study was to investigate the effects of PVI on the cardiac ANS. To exclude for the potential influence of sedatives and invasive procedures on the cardiac ANS, post-PVI neuECG recordings were conducted on the 2nd to 3rd day following the procedure, rather than on the day after the procedure. To match recording conditions, all neuECG recordings except pre-PVI in PerAF patients were required to be in sinus rhythm. Additionally, since restoration of sinus rhythm through electrical cardioversion may also induce changes in the cardiac ANS [14], electrical cardioversion was performed exclusively during the PVI procedure in PerAF patients, and post-PVI neuECG recordings were conducted while the patients were in sinus rhythm. We excluded two PAF patients before PVI because of artifacts or AF during neuECG recording. We also excluded 15 patients who required ablation in areas other than PVI (e.g., linear ablation, non-pulmonary vein foci ablation, and superior vena cava isolation) to observe the true effect of PVI. To minimize the influence of confounding factors, such as circadian rhythm, arrhythmia events, body position, and body temperature, 11 cases in which the patient’s condition after PVI was significantly different from before the procedure were excluded (e.g., febrile episodes, recording time of neuECG substantially different from before PVI, incessant atrial tachycardia [AT] or AF, and severe gastrointestinal motility disorder). We compared pre- and post-PVI SKNA parameters and their percentage change recorded from 61 eligible patients (PAF [n = 28] and PerAF [n = 33]). Additionally, we evaluated the impact of the PVI strategies on SKNA by comparing baseline and post-procedural values between the RFA and CBA groups. Antiarrhythmic drugs were discontinued after PVI in all patients, while other oral medications were continued. All patients were followed for at least 6 months after PVI. Post-PVI recurrence was defined as an AT or AF episode lasting > 30 s recorded on ECG or 24-h Holter ECG. Recurrence within 3 months of the blanking period was defined as early recurrence, and recurrence within 6 months thereafter was defined as distant recurrence.

We recorded skin sympathetic nerve activity (SKNA) from a total of 89 participants (paroxysmal atrial fibrillation [PAF], n = 49; persistent atrial fibrillation [PerAF], n = 40) one day before and 2–3 days after pulmonary vein isolation (PVI). Schema is an example of radiofrequency ablation. A total of 28 patients were excluded because they were thought to affect SKNA accuracy or the cardiac autonomic nervous system, resulting in a final enrollment of 61 eligible patients (PAF [n = 28], PerAF [n = 33]).

### 2.5. SKNA Recording and Analysis

In the present study, neuECG recordings were performed according to a previously reported experimental protocol in humans [5], and SGNA was estimated by measuring SKNA in the chest and upper extremities [5]. In canine models, sympathetic postganglionic fibers from the canine stellate ganglion have been shown to be distributed in the chest and upper extremities, a skin area between the level of the ipsilateral third cervical vertebra and the thirteenth rib [15]. The SKNA signals were recorded from the ECG patch electrodes in the chest wall (lead I) and were managed with LabChart Pro v8 software (ADInstruments Ltd., Bella Vista, Australia), with a sampling rate of 10 k/s [5]. The obtained signals were processed with a band-pass filter, and signals from 500 to 1000 Hz were displayed as SKNA. The average SKNA (aSKNA) was defined as the mean voltage of each signal. Fast Fourier transform (FFT) analyses of the integrated SKNA (iSKNA) were performed to collect frequency components (very low frequency [VLF]: 0.01–0.04 Hz, low frequency [LF]: 0.04–0.15 Hz, and high frequency [HF]: 0.15–0.40 Hz) [16,17]. The signals were downsampled from 10 k/s to 1 k/s before using FFT analyses with an FFT size of 128 k and an overlap of 93.75% to improve the frequency resolution. The normalization of the power of each frequency band was divided by the total power of the signals to obtain different frequency components (VLFnu, LFnu, and HFnu [normalized unit {nu}]). The dominant frequency was the frequency with the highest power in 30 min.

In general, SKNA amplitude analysis is more appropriate for evaluating instantaneous fluctuations in SNA. In contrast, frequency analysis reflects the relative balance of the cardiac ANS over a period of time. While VLF oscillations, LF oscillations, and LF/HF ratios become predominant with enhanced SNA, HF oscillations consistently represent background neural activity and correspond to the frequencies of normal respiration [17]. Typically, HF oscillations are overshadowed by the much larger burst activity of VLF and LF components.

### 2.6. Statistical Analysis

Data with non-normal distribution were reported as median (interquartile range [IQR]). Pearson’s chi-square test was conducted to compare categorical variables. Continuous variables between paired data, such as before and after PVI, were compared using the Wilcoxon rank–sum test, and unpaired data, such as those between PAF and PerAF groups, were compared using the Mann–Whitney test. Multiple regression analysis was conducted to compare the associations between multiple explanatory variables and SKNA. Spearman’s rank correlation coefficient was employed when comparing the strength of association between SKNA and echocardiographic results. All statistical analyses were conducted with JMP Pro version 14 (SAS Institute, Cary, NC, USA), and all figures were established using GraphPad Prism 9 (GraphPad Software, Inc., La Jolla, CA, USA). Two-sided *p*-values of <0.05 were considered statistically significant. As this study was a single-center study with a limited sample size, we used G*Power (version 3.1.9.7) to ensure sufficient post hoc power.

## 3. Results

### 3.1. Characteristics of the AF Patients

Table 1 shows characteristics of the AF patients. The median age of the PAF and PerAF groups was 70.0 (IQR: 59.5–73.8) and 64.0 (IQR: 56.5–74.0) years, respectively. The PerAF group demonstrated a significantly lower estimated glomerular filtration rate (eGFR) (63.3 [IQR: 54.1–69.1] vs. 75.2 [IQR: 69.9–84.4] mL/min/1.73m^2^, *p* < 0.001) and higher serum brain natriuretic peptide (BNP) levels (217.2 [IQR: 133.7–372.4] vs. 34.3 [IQR: 19.7–72.3] pg/mL, *p* < 0.001) compared to the PAF group. Echocardiographic results in the PerAF group demonstrated significantly lower LVEF (51.0 [IQR: 40.0–57.0] vs. 62.7 [IQR: 59.8–68.5]%, *p* < 0.001). Furthermore, although enlarged LAD (41.9 [IQR: 38.8–45.5] vs. 38.8 [IQR: 32.9–43.2] mm, *p* = 0.026) and LAVI (49.1 [IQR: 38.9–60.1] vs. 36.0 [IQR: 30.1–47.7] mL/m^2^, *p* = 0.014) were observed in the PerAF group, there were no differences in average E/e’ and peak tricuspid regurgitation velocity. Antiarrhythmic drugs and β-blockers were used in some patients before PVI; however, there were no differences between the two groups. The PVI strategy (RFA or CBA), procedure time, and fluoroscopy time were comparable between the two groups. Distant recurrence rates were similar, but early recurrence was significantly more frequent in the PerAF group (14 [42.4%] vs. 5 [17.9%], *p* = 0.039).

### 3.2. Representative SKNA Waveforms

**Figure 2:**
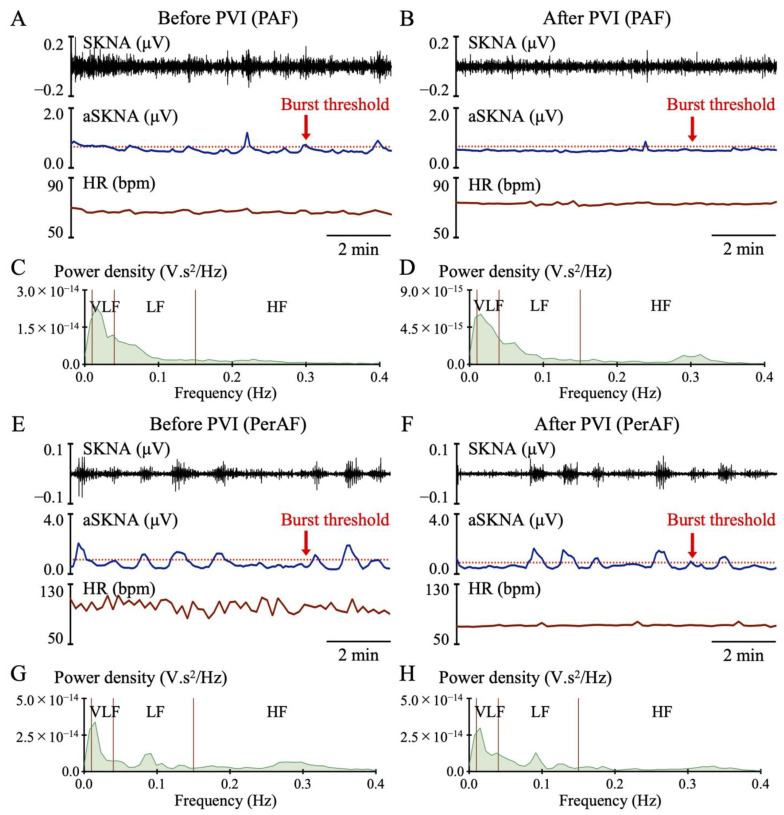
Representative skin sympathetic nerve activity (SKNA) waveforms. Among the waveforms obtained with neuECG, the top three are the raw SKNA waveform, the average SKNA (aSKNA) with burst threshold, and the heart rate (HR), respectively. The paroxysmal atrial fibrillation (PAF) group (**A**–**D**) demonstrated that SKNA amplitude, which was enhanced before ablation (**A**), attenuated after pulmonary vein isolation (PVI) (**B**). A mild increase in HR after PVI. Very low frequency (VLF) oscillations were dominant before PVI in the frequency distribution (**C**). Background high frequency (HF) oscillations became more pronounced after PVI (**D**). However, the persistent AF (PerAF) group (**E**–**H**) demonstrated decreased HR after PVI due to a return to sinus rhythm but with no difference in SKNA waveforms or frequency distribution.

Figure 2 illustrates representative SKNA waveforms and frequency distributions in both the PAF (Figure 2A–D) and PerAF (Figure 2E–H) groups. SKNA amplitude, which was enhanced before PVI, was attenuated after PVI in the PAF group. HR slightly increased after PVI. After PVI, the VLF oscillations were somewhat suppressed, and the background HF oscillations became more prominent in the frequency distribution. However, the PerAF group demonstrated minimal changes in SKNA waveforms and frequency distribution after PVI despite an HR reduction due to sinus rhythm restoration.

### 3.3. Differences in SKNA Depending on AF Type

**Figure 3:**
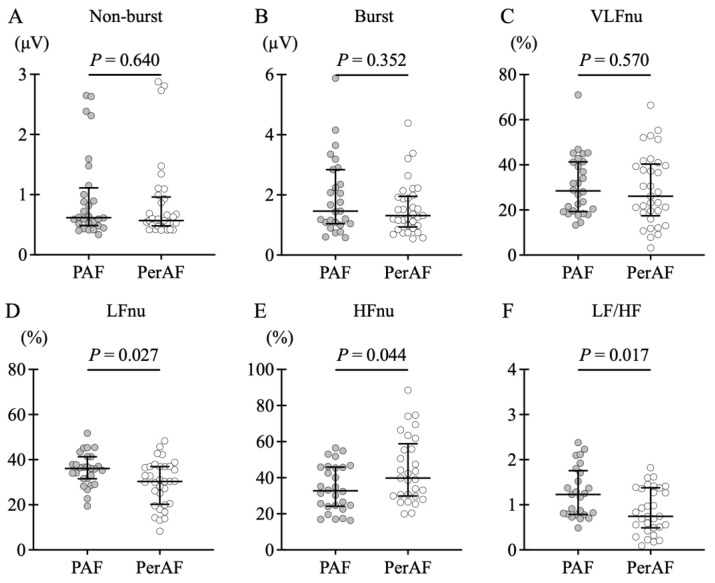
Comparison of skin sympathetic nerve activity (SKNA) before pulmonary vein isolation (PVI) in paroxysmal atrial fibrillation (PAF) and persistent AF (PerAF). A comparison of SKNA before PVI between the PAF and PerAF groups is described. The solid lines in the figure represent the median and interquartile range. No differences were found in (**A**) non-burst amplitude and (**B**) burst amplitude. Frequency analysis revealed no differences in the very low frequency (VLFnu [normalized units {nu}]) between groups (**C**). The PAF group had significantly higher low frequency (LFnu) (**D**) and significantly lower high frequency (HFnu) (**E**). In addition, the LF/HF ratio also showed a higher level in the PAF group (**F**).

Figure 3 shows the comparison of SKNA values at baseline before PVI. There were no obvious differences in SKNA non-burst amplitude and burst amplitude between the two groups. Frequency analysis showed no difference in VLFnu. However, LFnu was more enhanced in the PAF group (36.1 [IQR: 31.6–41.3] vs. 30.3 [IQR: 20.2–36.9]%, *p* = 0.027), and HFnu was more suppressed (32.7 [IQR: 24.2–45.9] vs. 39.8 [IQR: 29.9–58.9]%, *p* = 0.044). The LF/HF ratio also showed a higher level in the PAF group (1.23 [IQR: 0.79–1.76] vs. 0.74 [IQR: 0.49–1.38], *p* = 0.017).

### 3.4. SKNA Changes Caused by PVI

**Figure 4:**
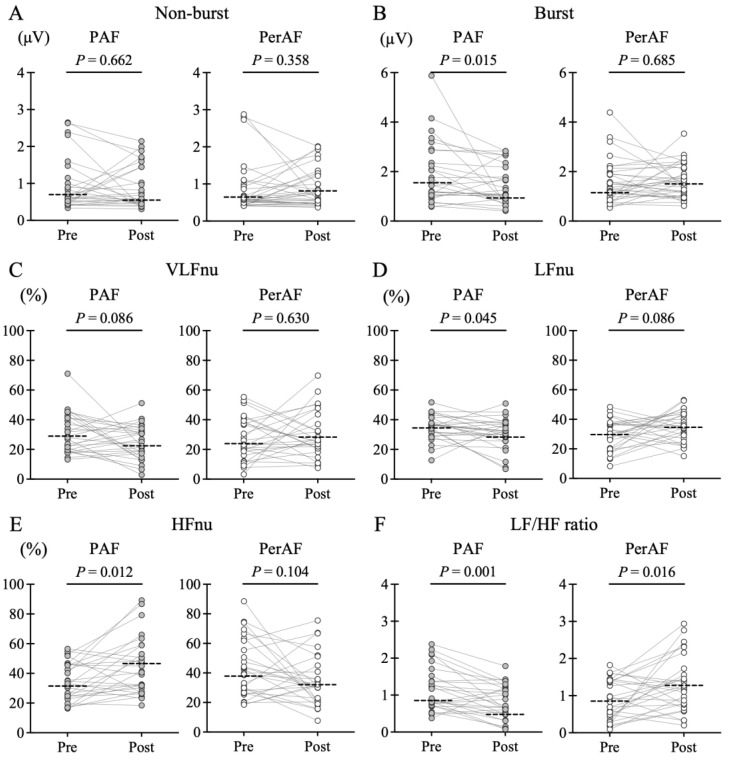
Changes in skin sympathetic nerve activity (SKNA) parameters before and after pulmonary vein isolation (PVI). A comparison of SKNA before and after PVI in paroxysmal atrial fibrillation (PAF) and persistent AF (PerAF) groups is shown, respectively. The dotted lines in the figure indicate the median value. No difference in non-burst amplitude was observed between the two groups (**A**), but a significant post-PVI reduction in burst amplitude was evident in the PAF group (**B**). No difference in very low frequency (VLFnu [normalized unit {nu}]) (**C**), but the low frequency (LFnu) decreased after PVI in the PAF group (**D**). High frequency (HFnu) was elevated, and the LF/HF ratio decreased in the PAF group (**E**,**F**), whereas the PerAF group demonstrated the opposite trend.

Figure 4 demonstrates the SKNA before and after PVI in the two groups. The PAF group was characterized by a significant attenuation in SKNA burst amplitude (1.46 [IQR: 1.04–2.84] vs. 1.09 [IQR: 0.78–2.17] μV, *p* = 0.015), LFnu (36.0 [IQR: 29.7–40.4] vs. 32.4 [IQR: 26.2–37.3]%, *p* = 0.045), and LF/HF ratio (0.91 [IQR: 0.73–1.52] vs. 0.71 [IQR: 0.48–1.21], *p* = 0.012), and a significant elevation in the HFnu (34.8 [IQR: 24.2–45.9] vs. 40.1 [IQR: 28.4–57.5]%, *p* = 0.015) after PVI. Conversely, such changes were unclear in PerAF, and rather, the LF/HF ratio elevated after PVI (0.88 [IQR: 0.44–1.40] vs. 1.25 [IQR: 0.76–1.69], *p* = 0.012). In the PAF group, LFnu decreased in the majority of patients after PVI (n = 20), while some patients showed an increase (n = 8). Although we confirmed the relationship between LF changes after PVI and patient characteristics, there were no clinical differences, such as echocardiographic findings or AF recurrence rate (Supplemental Table S1).

**Figure 5:**
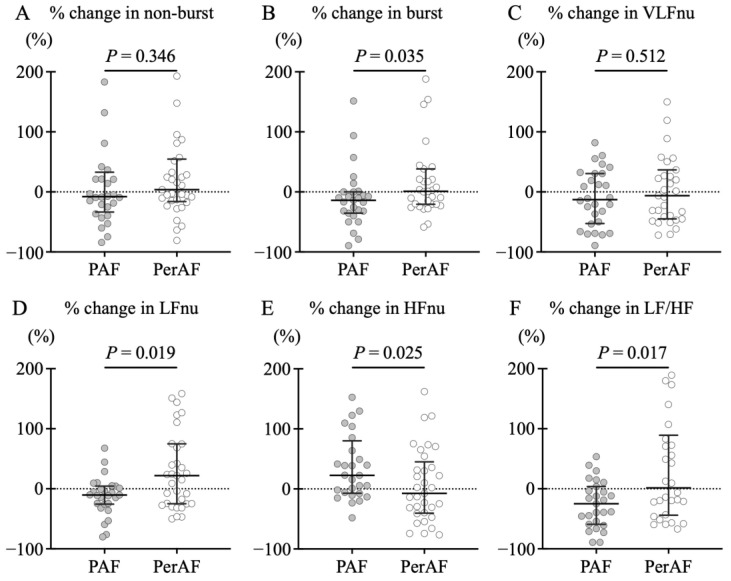
Comparison of the percentage changes in skin sympathetic nerve activity (SKNA) before and after pulmonary vein isolation (PVI). A comparison between paroxysmal atrial fibrillation (PAF) and persistent AF (PerAF) groups is described. The solid lines in the figure represent the median and interquartile range. No difference was found in (**A**) non-burst amplitude, but the PAF group demonstrated larger negative shifts from baseline in burst amplitude (**B**). Frequency analysis revealed no difference in the very low frequency (VLFnu [normalized unit {nu}]) between groups (**C**). The PAF group demonstrated significant negative shift in low frequency (LFnu) (**D**), positive shift in high frequency (HFnu) (**E**), and negative shift in LF/HF ratio (**F**).

Figure 5 illustrates the comparison of the percentage change in SKNA with PVI. The PAF group demonstrated larger negative shifts from baseline in burst amplitude of SKNA (−14.1 [IQR: −35.3–0.2] vs. 1.0 [IQR: −20.5–38.3]%, *p* = 0.035), LFnu (−10.4 [IQR: −25.7–4.5] vs. 21.9 [IQR: −24.9–74.9]%, *p* = 0.019), and LF/HF ratio (−24.9 [IQR: −59.3–3.6] vs. 1.6 [IQR: −44.0–89.2]%, *p* = 0.017) than the PerAF group. Additionally, HFnu in the PAF group exhibited a large positive shift from baseline (22.6 [IQR: −7.2–80.0] vs. −7.3 [IQR: −40.4–45.1]%, *p* = 0.025).

### 3.5. Comparison of PVI Strategies

The baseline characteristics of the RFA and CBA groups in PAF patients showed that the procedure time was shorter and the fluoroscopy time was longer in the CBA group. Efficacy was comparable, and echocardiographic findings showed no difference (Supplemental Table S2). Furthermore, no differences were detected between RFA and CBA in terms of SKNA changes based on the PVI strategies within the PAF group (Supplemental Figure S1).

### 3.6. Association Between SKNA and Echocardiographic Parameters

**Figure 6:**
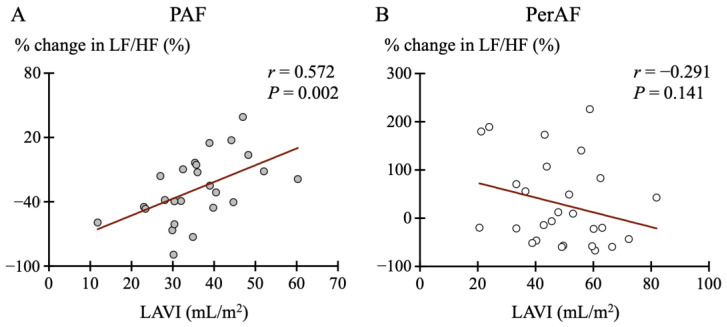
Correlation between the percentage change in the LF/HF ratio and left atrial volume index (LAVI). (**A**) A positive correlation between the percentage change in the LF/HF ratio and LAVI in the paroxysmal atrial fibrillation (PAF) group. (**B**) Such a relationship was not evident in the persistent AF (PerAF) group.

We evaluated the association with echocardiographic findings to investigate the mechanism underlying changes in SKNA after PVI. Table 2 presents the results of multiple regression analysis assessing the association between the percentage change in LF/HF ratio due to PVI and echocardiographic results. The PAF group demonstrated the strongest association with LAVI (*p* = 0.037), whereas the PerAF group exhibited unclear associations with all parameters. Spearman’s rank correlation coefficient revealed a significant positive correlation between the percentage change in the LF/HF ratio and LAVI in the PAF group (r = 0.572, *p* = 0.002) (Figure 6). In particular, the LF/HF ratio decreased more substantially post-PVI in cases with smaller LAVI. No such clear association was found in the PerAF group.

### 3.7. Post Hoc Power of the Present Study

Given the small sample size, it is necessary to estimate whether the present study has adequate power to detect a true association between SKNA and the neuromodulatory effects of PVI. Among the results determined to be statistically significant, the post hoc power when performing the Wilcoxon rank–sum test with a significance level of 0.05 was as follows: Comparing SKNA burst amplitude and LF/HF ratio before and after PVI in the PAF patients yielded effect sizes of 0.45 and 0.62 and post-hoc power of 0.61 and 0.86, respectively. Furthermore, when comparing the percentage changes in SKNA burst amplitude and LF/HF ratio before and after PVI between the PAF and the PerAF patients, the effect size was 0.65 and 1.61, and the post hoc power was 0.68 and 0.99, respectively. These show that our sample size was adequate, and the present study was sufficiently powered to detect the true association.

## 4. Discussion

### 4.1. Major Findings

To the best of our knowledge, this is the first study to investigate the acute effects of PVI on SKNA and to compare PAF and PerAF patients. The primary results of the present study indicate that SKNA was more enhanced in PAF patients and PVI significantly reduced burst amplitude and LF/HF ratio, but not in PerAF patients. Furthermore, LA enlargement indicated attenuating PVI-induced SKNA reduction in PAF patients. These results support our hypothesis that LA remodeling progression in patients with AF inhibits PVI-mediated neuromodulation.

### 4.2. Cardiac Autonomic Disorders in AF

A bidirectional relationship likely exists between cardiac ANS and AF [18], as supported by numerous studies [3,4,6,7,18,19,20,21,22,23]. Previous research has demonstrated a strong association between abnormal autonomic innervation and AF in animal and human models. Canine models with pacing-induced AF exhibited increased atrial sympathetic innervation [21]. Additionally, simultaneous sympathetic–parasympathetic activation has been observed to be the main trigger of AF [3]. AF results in atrial sympathetic and parasympathetic hyperinnervation and heterogeneity, increasing AF vulnerability [23]. Although most studies investigating AF and ANS have primarily focused on efferent autonomic inputs to the heart [19], it is becoming clear that AF can also disrupt cardiac afferent reflexes [22]. These findings support the concept of autonomic disorder as a key pathophysiological factor in AF. However, there are few studies that assess differences in the cardiac ANS according to the type of AF. In the present study, a higher level of SKNA enhancement was particularly observed in PAF patients. One possible reason is that PerAF patients generally have higher BNP levels. BNP exerts an inhibitory effect on SNA through the renin–angiotensin system [24], and thus, SKNA in PerAF patients might have been adjusted in an inhibitory direction. However, the difference between both groups is not extremely large, and despite the elevated BNP levels, it could be interpreted as insufficient SNA inhibition in PerAF patients. Furthermore, in PerAF patients, the effects of AF may have led to a shift in the set point of SNA compared to when sinus rhythm was maintained.

A current challenge is the low reliability of ANS assessment during AF. Existing methods fail to adequately account for subtle baroreceptor sensitivity and pulse rate fluctuation during AF. Muscle SNA (MSNA) is one of the tools that can be measured during AF. Although single-unit MSNA has been shown to increase during AF [25], concerns remain regarding its invasiveness. SKNA, being non-invasive and independent of pulse rate fluctuation, may serve as a suitable tool for SNA evaluation during AF. Notably, enhanced SKNA during AF has been linked to increased ventricular rate responses [6].

### 4.3. PVI-Mediated Neuromodulation

A previous meta-analysis revealed that although GP-targeted catheter ablation reduces AF recurrence, its efficacy in PerAF is limited [26]. The relationship between PVI and GP remains unclear, and various studies have investigated PVI-mediated neuromodulation. A study of MSNA revealed that PVI significantly decreased MSNA in patients with AF [12]. Heart rate variability (HRV) studies have observed a decrease in LF, HF, and LF/HF ratio with a transient increase in HR after PVI. Further, a decrease in LF/HF ratio was associated with arrhythmia prognosis [27]. Collectively, these reports indicate that PVI-mediated neuromodulation caused a denervation effect on cardiac ANS. Similarly, the present study demonstrated a decrease in the LF/HF ratio after PVI in PAF patients. In frequency analysis, most PAF patients showed a decrease in LF oscillations after PVI, whereas some patients exhibited an increase. However, no clinical differences, such as AF recurrence rate, were observed with or without reduction of LF oscillation. Since frequency analysis reflects the balance of the cardiac ANS, the LF/HF ratio might be a more reliable indicator than the individual components. It is generally observed that transient increases in HR occur after PVI; however, SNA is not an independent factor regulating HR. The autonomic nerve endings surrounding the heart contain both sympathetic and parasympathetic fibers. While SKNA reflects only SNA, a previous study has shown that baroreflex sensitivity (BRS), which reflects parasympathetic function, decreases after PVI [11]. Therefore, the increase in HR after PVI may be influenced by a complex interaction between sympathetic and parasympathetic nerves.

The neuromodulatory effects of PVI may vary depending on the physiological characteristics or anatomical background of the individual patient’s heart and the details of the ablation procedure. As an example, a previous study using SKNA reported that SKNA increased one day after PVI and then returned to baseline 3 months later [28]. Interestingly, it was also reported that the neuromodulatory effects during the acute phase of PVI differ depending on the energy setting in RFA [29]. This is inconsistent with our results and may be attributed to methodological differences such as the sedation method, PVI strategy, and SKNA measurement conditions. Particularly on the day following PVI, it is speculated that SNA was more easily activated due to various factors such as invasiveness. Although the sample size is small, we confirmed that there was no significant difference in the neuroregulatory effects between RFA and CBA. This finding aligns with previous studies showing no difference in HRV changes between PVI strategies [30,31], as well as with meta-analysis results demonstrating equivalent clinical outcomes [32]. Lesions visualized by late gadolinium-enhanced magnetic resonance imaging are more extensive and continuous in CBA compared to RFA; however, the lesion depth could not be fully assessed [33]. The difference in HR increases between PVI strategies may be attributed to variations in parasympathetic nerve influences.

### 4.4. Impairment of Neuromodulation Due to LA Remodeling

One of the important results of the present study is the positive correlation between PVI-induced SKNA reduction and LAVI in PAF patients. Atrial electrical and structural remodeling leads to conduction abnormalities and atrial myocardial dysfunction and forms a substrate for AF [34]. Neural remodeling has been demonstrated in a canine AF model [35], suggesting that AF may alter the cardiac ANS. These results imply that cardiac ANS dysfunctions are implicated not only in the triggering and sustaining of AF but also in the development of AF-induced ANS disorders.

PVI reduces BRS, whereas this effect is attenuated in PerAF with LA remodeling [11]. Furthermore, PVI significantly reduced MSNA, and this reducing effect was observed to be positively correlated with the post-PVI reduction rate of LAVI [12]. PerAF with enlarged LAVI is predicted to have advanced neural remodeling, and the denervation effect of PVI may be attenuated. Additionally, other mechanisms may be involved in the relationship between LA remodeling and the neuromodulatory effects of PVI. Chronic inflammation and oxidative stress in the atrial muscle are known to cause degeneration [36]. Furthermore, increased fibrous tissue within the LA may alter autonomic innervation, affecting SKNA. Because the mechanism of LA remodeling is complex and the progression process is not uniform, further long-term studies are needed to determine how differences in the effects on the cardiac ANS relate to arrhythmia prognosis.

### 4.5. Study Limitations

First, this is a single-center study with a limited sample size of Asian participants. Second, although SKNA may be less affected, accurate assessment of cardiac ANS during AF is still difficult. Even after electrical cardioversion in PerAF patients, baroreflex function may remain impaired for several days [14]. SKNA is an assessment tool for SGNA and should not be used to describe parasympathetic activity. Furthermore, there is a lack of knowledge as to whether PVI primarily affects the sympathetic efferent or afferent pathway. We need to continue investigating how SKNA reflects efferent or afferent cardiac SNA and what significance SKNA has during AF.

The present study included patients using β-blockers. It is possible that baseline central SNA could increase as a feedback effect [37]. However, there was no difference in the frequency of β-blocker use between the two groups, and no alterations in medication were made prior to or following the PVI procedure. Therefore, the potential impact of β-blocker use on the results is likely to be minimal.

Because the cardiac ANS can be influenced by a variety of factors [38], including circadian rhythm, arrhythmia, body position, body temperature, medications, and heart failure, efforts must be made to minimize confounding factors. Although we matched testing conditions as much as possible, neuECG recordings were limited to 30 min per day. The present study focused exclusively on acute changes in SKNA after PVI. We recognize this study as a pilot study, and in the future, it would be desirable to conduct a larger study in which the protocol includes a long-term continuous recording of neuECG and a longer observation period. Additional investigation is required into how the neuromodulatory effects of PVI and differences in BNP levels affect arrhythmia prognosis after PVI. As an interventional study to observe the effects of PVI, it is also important to consider a study design that includes AF patients who did not undergo PVI as a control group.

## 5. Conclusions

The present study revealed that PVI significantly decreased SKNA in PAF patients but not in PerAF. The LA enlargement in PAF patients exhibited attenuating PVI-induced SKNA reduction. These results indicate that LA remodeling progression inhibits PVI-mediated neuromodulation.

## Figures and Tables

**Table 1:** Characteristics of the AF patients.

Variables	PAF (n = 28)	PerAF (n = 33)	*p*-Value
Age (years)	70.0 (59.5–73.8)	64.0 (56.5–74.0)	0.365
Female sex, n (%)	20 (71.4)	21 (63.4)	0.518
BMI (kg/m^2^)	23.4 (21.7–25.4)	24.1 (21.7–26.3)	0.572
eGFR (mL/min/1.73 m^2^)	75.2 (69.9–84.4)	63.3 (54.1–69.1)	<0.001
BNP (pg/mL)	34.3 (19.7–72.3)	217.2 (133.7–372.4)	<0.001
Echocardiographic results			
LVEF (%)	62.7 (59.8–68.5)	51.0 (40.0–57.0)	<0.001
LAD (mm)	38.8 (32.9–43.2)	41.9 (38.8–45.5)	0.026
LAVI (mL/m^2^)	36.0 (30.1–47.7)	49.1 (38.9–60.1)	0.014
Average E/e’ ratio	8.4 (7.4–12.0)	8.8 (7.4–11.8)	0.884
Peak TR velocity (m/s)	2.3 (2.1–2.6)	2.2 (1.9–2.4)	0.113
Medications before PVI			
AADs, n (%)	6 (21.4)	3 (9.1)	0.178
β-blockers, n (%)	13 (46.4)	18 (54.6)	0.528
Ablation results			
RFA, n (%)	18 (64.3)	24 (72.7)	0.487
CBA, n (%)	10 (35.7)	9 (27.3)	0.487
Procedure time (min)	137.5 (121.3–157.5)	145.0 (119.0–175.0)	0.357
Fluoroscopy time (min)	15.7 (12.1–23.6)	18.5 (10.6–23.3)	0.983
Early recurrence, n (%)	5 (17.9)	14 (42.4)	0.039
Distant recurrence, n (%)	1 (3.6)	3 (9.1)	0.386

Data are presented as median (interquartile range) or total number (%). AAD, antiarrhythmic drug; AF, atrial fibrillation; BMI, body mass index; BNP, brain natriuretic peptide; CBA, cryoballoon ablation; eGFR, estimated glomerular filtration rate; LAD, left atrial diameter; LAVI, left atrial volume index; LVEF, left ventricular ejection fraction; PAF, paroxysmal atrial fibrillation; PerAF, persistent atrial fibrillation; PVI, pulmonary vein isolation; RFA, radiofrequency ablation; TR, tricuspid regurgitation.

**Table 2:** Multiple regression analysis between the percentage change in LF/HF ratio and echocardiographic results.

Variables	Estimate	SE	*t*-Value	*p*-Value
PAF				
LVEF (%)	1.981	1.017	−1.95	0.073
LAD (mm)	1.648	1.351	1.22	0.244
LAVI (mL/m^2^)	1.599	0.690	2.32	0.037
Average E/e’ ratio	0.851	2.476	0.34	0.736
Peak TR velocity (m/)	25.302	16.331	0.34	0.145
PerAF				
LVEF (%)	−0.856	5.279	−0.16	0.873
LAD (mm)	−16.170	15.631	−1.03	0.315
LAVI (mL/m^2^)	−4.891	4.664	−1.05	0.308
Average E/e’ ratio	14.293	11.455	1.25	0.228
Peak TR velocity (m/s)	−27.220	155.754	−0.17	0.863

LAD, left atrial diameter; LAVI, left atrial volume index; LVEF, left ventricular ejection fraction; PAF, paroxysmal atrial fibrillation; PerAF, persistent atrial fibrillation; TR, tricuspid regurgitation; SE, standard error.

## Data Availability

The data presented in the study are available on request from the corresponding author due to participant privacy protection.

## Supplementary Materials

The following supporting information can be downloaded at: https://www.mdpi.com/article/10.3390/jcdd12040123/s1. Figure S1. Differences in the percentage change in skin sympathetic nerve activity (SKNA) by the pulmonary vein isolation (PVI) strategies in the paroxysmal atrial fibrillation (PAF). A comparison between radiofrequency ablation (RFA) and cryoballoon ablation (CBA) is presented. The solid lines in the figure indicate the median and interquartile range. No difference was found between (A) the non-burst amplitude and (B) the bust amplitude of SKNA. The frequency analysis revealed no difference in the percentage change in (C) very low frequency (VLFnu [normalized unit {nu}]), (D) low frequency (LFnu), (E) high frequency (HFnu), and (F) LF/HF ratio. Table S1. Relationship between PVI-mediated LFnu changes and patient characteristics for the PAF patients. Table S2. Characteristics of RFA and CBA groups in the PAF patients.
