# Supplementary material for: Effects of Pulmonary Vein Isolation for Atrial Fibrillation on Skin Sympathetic Nerve Activity in Association with Left Atrial Remodeling
Source: J Cardiovasc Dev Dis. 2025 Mar 30;12(4):123. doi: 10.3390/jcdd12040123 (PMC12028141; doi:10.3390/jcdd12040123)
Supplement: Supplementary file 1 [file jcdd-12-00123-s001.zip › jcdd-3543511-supplementary.pdf]

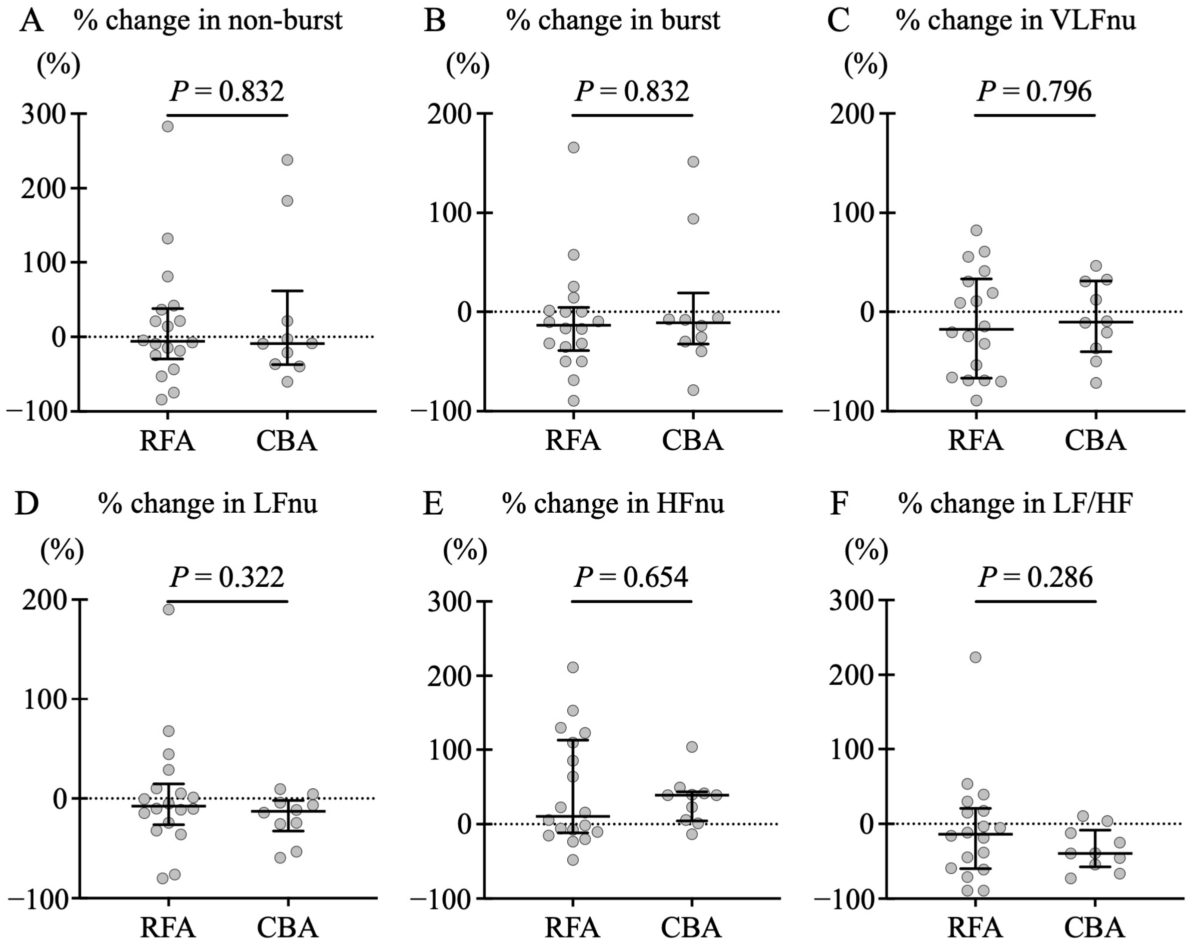

Figure S**1.** Differences in the percentage change in skin sympathetic nerve activity (SKNA) by the pulmonary vein isolation (PVI) strategies in the paroxysmal atrial fibrillation (PAF).

A comparison between radiofrequency ablation (RFA) and cryoballoon ablation (CBA) is presented. The solid lines in the figure indicate the median and interquartile range. No difference was found between **(A)** the non-burst amplitude and **(B)** the bust amplitude of SKNA. The frequency analysis revealed no difference in the percentage change in **(C)** very low frequency (VLFnu [normalized unit {nu}]), **(D)** low frequency (LFnu), **(E)** high frequency (HFnu), and **(F)** LF/HF ratio.

Table S1. Relationship between PVI-mediated LFnu changes and patient characteristics for the PAF patients

Variables	Decreased LFnu (n = 20)	Increased LFnu (n = 8)	*P*-value
Age (years)	71.5 (62.3–73.8)	68.5 (56.8–73.3)	0.415
Female sex, n (%)	5 (25.0)	3 (37.5)	0.508
BMI (kg/m^2)	23.4 (21.3–26.0)	24.2 (21.7–25.3)	0.593
eGFR (mL/min/1.73m^2)	75.1 (69.9–85.6)	77.9 (66.3–82.6)	0.978
BNP (pg/mL)	30.7 (19.7–106.5)	41.9 (20.7–58.2)	1.000
Echocardiographic results			
LVEF (%)	62.7 (59.8–69.3)	63.5 (55.5–68.3)	0.760
LAD (mm)	38.8 (32.8–42.9)	39.5 (33.5–45.0)	0.576
LAVI (mL/m^2)	35.4 (29.9–46.8)	45.6 (36.2–50.2)	0.215
Average E/e’ ratio	10.3 (6.8–11.8)	8.1 (7.4–12.6)	1.000
Peak TR velocity (m/sec)	2.3 (2.1–2.6)	2.3 (2.1–2.5)	0.537
Medications before PVI			
AADs, n (%)	8 (40.0)	5 (62.5)	0.281
β-blockers, n (%)	6 (30.0)	0 (0.0)	0.081
Ablation results			
Procedure time (min)	137.5 (116.3–157.5)	137.5 (130.0–162.3)	0.665
Fluoroscopy time (min)	15.8 (12.9–27.8)	12.8 (10.1–21.2)	0.204
Early recurrence, n (%)	4 (20.0)	1 (12.5)	0.639
Distant recurrence, n (%)	3 (15.0)	0 (0.0)	0.246

Data are presented as median (interquartile range) or total number (%). AAD, antiarrhythmic drug; BMI, body mass index; BNP, brain natriuretic peptide; eGFR, estimated glomerular filtration rate; LAD, left atrial diameter; LAVI, left atrial volume index; LF, low frequency; LVEF, left ventricular ejection fraction; nu, normalized unit; PAF, paroxysmal atrial fibrillation; PVI, pulmonary vein isolation; TR, tricuspid regurgitation

Table S2. Characteristics of RFA and CBA groups in the PAF patients

Variables	RFA (n = 18)	CBA (n = 10)	*P*-value
Age (years)	71.5 (64.3–74.0)	69.0 (55.3–73.0)	0.400
Female sex, n (%)	5 (27.8)	3 (30.0)	0.901
BMI (kg/m^2)	23.2 (20.9–25.1)	24.1 (22.1–27.8)	0.281
eGFR (mL/min/1.73m^2)	76.1 (70.2–83.7)	74.6 (61.8–86.4)	0.684
BNP (pg/mL)	49.0 (24.2–148.6)	28.2 (15.4–50.8)	0.108
Echocardiographic results			
LVEF (%)	62.2 (58.0–69.6)	64.0 (60.0–67.1)	0.701
LAD (mm)	40.7 (32.6–45.6)	38.0 (33.4–40.3)	0.533
LAVI (mL/m^2)	38.9 (28.1–52.1)	35.5 (30.3–41.9)	0.760
Average E/e’ ratio	8.3 (7.4–12.3)	10.3 (6.1–11.6)	0.744
Peak TR velocity (m/sec)	2.3 (2.1–2.6)	2.4 (2.2–2.8)	0.432
Medications before PVI			
AADs, n (%)	6 (21.4)	3 (9.1)	0.178
β-blockers, n (%)	3 (16.7)	3 (30.0)	0.410
Ablation results			
Procedure time (min)	142.5 (133.8–165.8)	115.0 (98.8–132.5)	0.003
Fluoroscopy time (min)	12.8 (10.4–16.6)	25.0 (15.9–28.9)	0.001
Early recurrence, n (%)	2 (11.1)	3 (30.0)	0.211
Distant recurrence, n (%)	3 (16.7)	0 (0.0)	0.172

Data are presented as median (interquartile range) or total number (%). AAD, antiarrhythmic drug; BMI, body mass index; BNP, brain natriuretic peptide; CBA, cryoballoon ablation; eGFR, estimated glomerular filtration rate; LAD, left atrial diameter; LAVI, left atrial volume index; LVEF, left ventricular ejection fraction; PAF, paroxysmal atrial fibrillation; PVI, pulmonary vein isolation; RFA, radiofrequency ablation; TR, tricuspid regurgitation
